# Plasmid copy number as a modulator in bacterial pathogenesis and antibiotic resistance

**DOI:** 10.1038/s44259-025-00145-9

**Published:** 2025-08-18

**Authors:** Helen Wang, Enrique Joffré

**Affiliations:** 1 Department of Medical Biochemistry and Microbiology, Uppsala University, Uppsala, Sweden; 2 Department of Microbiology, Tumor and Cell Biology, Karolinska Institute, Stockholm, Sweden

**Keywords:** Clinical microbiology, Microbial genetics, Pathogens, Microbiology, Diseases, Translational research

## Abstract

Plasmid copy number (PCN), the average number of plasmids per bacterial cell, links gene dosage to key traits such as host fitness, virulence, antibiotic resistance and evolutionary potential. Although often viewed as static, PCN is a dynamic, regulated trait responsive to environmental cues and selection pressures. This review examines the regulatory mechanisms controlling PCN and its impact on the trade-offs between bacterial fitness, virulence cost, and antibiotic resistance.

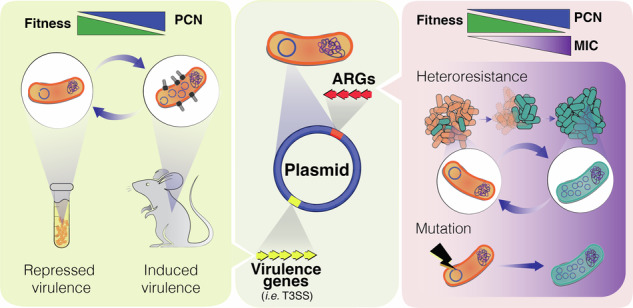

## Introduction

Plasmids are extrachromosomal DNA elements that exemplify bacterial adaptability by harboring genes for antibiotic resistance, virulence, metabolic versatility, and stress tolerance across species boundaries^1^. They replicate independently of the chromosome under tight feedback control, typically undergoing one replication per cell cycle, with deviations sensed and corrected to maintain a stable copy number^2^. Although plasmid-encoded traits can greatly enhance bacterial survival under antibiotic or host-immune pressure, their carriage incurs a metabolic cost on the bacterial host^3^. Beyond integrating precise copy-number regulation with partitioning and toxin-antitoxin systems to ensure faithful inheritance while minimizing fitness costs^4^, plasmid persistence is also shaped by diverse ecological and evolutionary dynamics. These include efficient infectious transmission (conjugative plasmids), variable fitness effects across host genetic backgrounds, interactions with other plasmids, source-sink spillover transmission from proficient to less-proficient host species, host compensatory evolution to alleviate fitness costs, and the ‘piggybacking’ of plasmids on host adaptations to new niches (reviewed in Brockhurst and Harrison^5^).

Resistance and virulence genes often coexist on the same plasmid, and acquisition of a single element can transform a benign strain into a life-threatening pathogen^6^. Understanding how PCN dynamics balance fitness costs with the benefits of increased gene dosage is therefore crucial, not only for unraveling bacterial evolution, but also for developing strategies to limit the rise of untreatable infections.

In this review, we present current knowledge on the regulation and maintenance of plasmid copy number (PCN) mechanisms and examine how dynamic changes in PCN impact: (i) bacterial fitness trade‑offs; (ii) virulence gene dosage and pathogenesis; (iii) the emergence and heterogeneity of antibiotic resistance; and (iv) long‑term evolutionary trajectories via mutation supply and segregational drift. Our goal is to provide an integrated, mechanistic overview on PCN as an evolvable trait, highlighting both the molecular circuitry (iteron‑, RNA‑, and protein‑mediated control, partitioning and toxin–antitoxin systems) and the population‑level consequences (fitness costs, antibiotic heteroresistance, and evolutionary dynamics). By bridging these perspectives, we aim to underscore the pivotal role of PCN in bacterial adaptation and identify critical gaps for future investigation.

## Regulation of Plasmid Copy Number

Accurate segregation of plasmids into daughter cells after cell division relies on autonomous maintenance and partitioning mechanisms, which vary depending on the plasmid copy number. The PCN refers to the number of plasmid copies within a single bacterial cell and it varies with plasmid types and the host background^7^. Typically, low-copy-number plasmids (LCP) are larger, while medium-copy-number plasmids (MCP) are smaller. LCP often present at 1–5 copies per cell, depend on tightly regulated partitioning systems to ensure stable inheritance. These systems avoid plasmid loss during cell division while maintaining a low metabolic burden on the host^2^. In contrast, high-copy-number plasmids (HCP), which are present at 50–100 copies per cell, replicate more frequently, and are generally segregated randomly^8^. Due to their high yield, HCPs are commonly used in molecular biology for cloning vectors and protein production.

To ensure plasmid stability and minimize host metabolic burden, bacteria employ tightly regulated systems for both plasmid replication-control and partitioning. Here, we briefly outline the classical and modern classifications of bacterial plasmids and explore the principal mechanisms that govern PCN and ensure faithful plasmid inheritance during cell division (Table 1).

**Table 1:** Summary of key features of representative plasmids from the major incompatibility groups

Inc group	Rep type	Representative plasmid	Size (kb)	PCN	Host range	Associated traits	PCN regulation mechanism	Refs
IncF	RepAFII	F (F-factor)	95–100	1–3	Narrow	AMR (e.g. *bla*_TEM_, *tetA*); virulence	Combination: Antisense RNA CopA with repressor CopB limit RepA synthesis (IncFII R1)	^76^
IncI	RepI1	R64 (*Shigella* R plasmid)	~120	1–3	Narrow	AMR (often ESBL β-lactamases in *E. coli/Salmonella*)	Antisense RNA: Small Inc RNA binds repZ mRNA to block Rep protein translation	^77^
IncP	TrfA	RK2/RP4	~60	4–7	Broad (Gram–negative)	MDR AMR; heavy‑metal resist.	Iteron (TrfA–oriV)	^78^
IncQ	RepA_Q_	RSF1010 (a.k.a. R1162)	8.7	~10–20 (in *E. coli*)	Broad (Gram–negative)	AMR (streptomycin + sulfonamide)	No iteron or antisense system; three Rep proteins (RepA, -B, -C) with multiple promoters ensure replication across hosts	^79^
IncN	RepN	pKM101 (derivative of R46)	~54	~2–5 (low)	Broad (Gram–negatives)	AMR (e.g. quinolone (*qnr*), β-lactamases)	Iteron-based: Rep initiator binds repeated oriV sites; classic iteron copy-control	^80^
IncW	RepW	R388 (*E. coli* R-plasmid)	33	1–2	Broad (many Alphαproteobacteria)	AMR (trimethoprim resistance)	Iteron-based: RepA initiator with ~5 iteron repeats at oriV (classic θ-replication control)	^81^
IncH	RepH	R27	180	1–2	Medium (Enterobacteriaceae)	AMR (MDR R-plasmid: e.g. chloramphenicol, sulfonamides); heavy metal resistance (arsenic, tellurite, etc.)	Iteron-based: Often carries dual replicons (e.g. two IncHI replicons) with iteron-regulated Rep proteins	^82^
IncA/C	RepA_A/C_	pRA1	140–180	1–2	Broad (enteric bacteria, *Vibrios*, etc.)	AMR (multiple, e.g. *bla*_NDM-1_, *bla*_CMY-2,_ metallo-β-lactamases)	Iteron-based: Encodes RepA with ~13 direct repeats; RepA-iteron binding autoregulates initiation	^83^
ColE1	RNA‑mediated	ColE1 / pBR322 (lab vector)	~6.6	~15–24	Narrow (*E. coli* and close relatives)	Bacteriocin (colicin E1 toxin) production; cloning vectors carry AMR markers	Antisense RNA: RNA I binds the replication primer RNA II, with Rom/Rop protein stabilizing the RNA–RNA duplex to inhibit initiation	^84^

### Plasmid classification

Plasmid incompatibility (Inc) groups were historically defined by the inability of two plasmids with similar replication control mechanisms to stably coexist in the host cell. In other words, if two plasmids competed for the same replication machinery and could not be maintained together, they were regarded incompatible and assigned to the same Inc group^9^. Thus, plasmids in a given Inc group typically share high similarity in their replication and/or partitioning systems. This labor-intensive classification relied on mating or transformation experiments to test co-maintenance of plasmids (reviewed in Norvick et al.^10^). In 1988, Counturier et al.^11^ introduced a molecular approach to Inc typing, using cloned replicon DNA probes for hybridization with plasmid samples. This method laid the foundation for the development of PCR-based replicon typing (PBRT) method in 2005, which streamlined Inc group classification. PBRT defined multiple Inc groups (IncF, IncI, IncN, IncA/C, etc.), often further subdivided by specific replicons (e.g., IncFIA, FIB, FIC, FII)^12^. These replicon-based Inc designations remain widely used, particularly for classifying plasmids in Enterobacteriaceae.

However, traditional Inc/replicon typing is limited by its single-locus focus, because plasmids with divergent or mosaic replicons often escape detection, and plasmids with multiple replicons can complicate Inc group assignment. To address this, in silico tools such as PlasmidFinder have been developed to automate Inc typing in large datasets by detecting known replicon sequences from genomic data. Nevertheless, such tools cannot classify plasmids lacking homology to known replicons^13^. A recent and promising approach involves the entire plasmid comparisons using clustering and average nucleotide identity methods to defines plasmid taxonomic units (PTUs)^14^. The COPLA tool implements this approach within a universal, species-independent framework, available both as an automatable pipeline and a web service, enabling rapid and standardized plasmid classification while capturing broader evolutionary relationships often missed by traditional methods^15^. However, PTUs may fail to resolve small cryptic plasmids and those do not clearly correspond to legacy Inc groups^16^. As a complementary approach, relaxase (MOB) typing, implemented in the MOB-suite, offers a species-wide plasmid classification system (reviewed in^17^). This method categorizes plasmids by their conjugative relaxase gene and provide key information of the plasmid’s mobilization potential. In combination with detection of mating pair formation genes (i.e., type IV secretion system), it can distinguish between self-transmissible or mobilizable plasmids^18^.

### Replication-based PCN control

Bacteria regulate PCN through three main replication-based strategies: iteron binding, antisense RNA, and combined RNA-protein repression mechanisms^19,20^. These systems act through feedback loops and adjust replication initiation in response to copy number fluctuations to ensure a steady-state PCN.

**Fig. 1:**
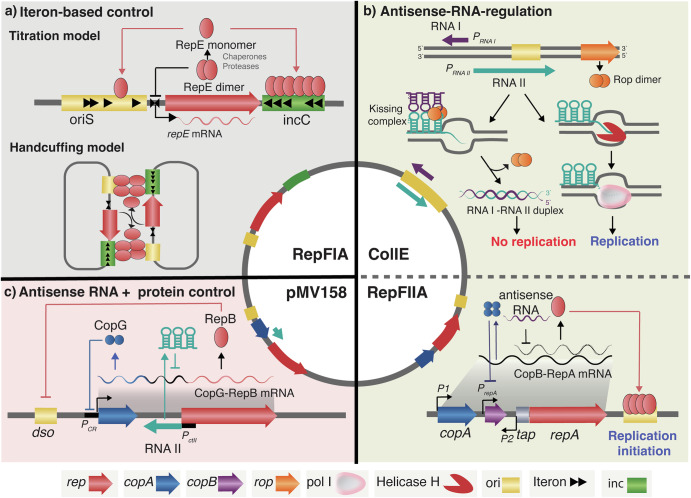
Schematic comparison of major PCN control mechanisms. Inner plasmids illustrate the gene organization of minimal regulatory units for each mechanism. **a** Iteron‑based control: A minimal iteron‑regulated replicon contains an origin of replication (ori) with multiple direct repeats (iterons) that specifically bind Rep monomers. Additional iterons downstream of the *rep* gene modulate PCN via two non–mutually exclusive mechanisms: *titration*, where excess iterons sequester free Rep monomers and reduce initiation; and *handcuffing*, where Rep–iteron complexes from different plasmid molecules interact, forming “handcuffs” that block Rep assembly at the ori^6^. **b** ColE1‑type antisense RNA control (**upper part**): The ColE1 plasmid ori drives transcription of an RNA pre‑primer (RNA II), which hybridizes to the template DNA forming R‑loop, and is processed by RNase H, allowing replication to initiate by DNA Pol I. Copy number is limited by RNA I, a 108 bp antisense RNA transcribed convergently. RNA I rapidly base‑pairs with RNA II, aided by the Rop protein, preventing R‑loop formation and primer maturation. Fast turnover of RNA I ensures dynamic regulation in response to physiological conditions^4^. RepFIIA plasmid (**lower part**): Replication initiates at the ori site downstream of the *repA* gene, whose translation is coupled to the upstream *tap* leader peptide. A weak constitutive promoter, P1, drives transcription of the *copB–tap–repA* operon (copB-repA-mRNA), while a stronger downstream promoter, P2, is kept inactive by CopB repressor binding. CopA antisense RNA inhibits *tap* translation, indirectly reducing RepA synthesis. **c** Combined protein–RNA control in pMV158. In the streptococcal plasmid pMV158, *repB* (encoding initiator) and *copG* (encoding repressor) form an operon driven by promoter *pcr*. CopG protein binds *pcr* to auto‑repress *repB* transcription. A second promoter, *pctII*, produces a small antisense RNA (ctRNA II), which binds the leader region of the *copG‑repB* mRNA, blocking RepB translation. Together, CopG and ctRNA II regulate PCN via coordinated transcriptional and translational repression of the replication initiator^4,85^.

- **Iteron-based control**: Iterons are short direct repeats at the origin of replication that bind Rep initiator proteins. At low Rep concentrations, Rep-iteron bindings trigger replication initiation. As PCN rises, additional Rep molecules are sequestered by iterons in trans (“handcuffing”), inhibiting initiation and preventing over-replication (Fig. 1a). This negative feedback loop mechanism is especially common in low-copy theta-replicating plasmids such as P1 and F^2^. Loftie-Eaton and Rawlings^21^ demonstrated that adding a 6-bp iteron repeat resulted in increased PCN, while a 22-bp repeat reduced PCN. Hence, variation in iteron numbers in IncQ plasmids significantly alters PCN, suggesting the critical role of iteron count in PCN control.
- **Antisense RNA-based control**: Many plasmids encode a small cis-encoded antisense RNA (ctRNA) that hybridize with a complementary leader on the Rep-encoding mRNA or primer precursor, thereby blocking primers maturation, terminating RNA synthesis, or preventing translation of replication-related genes. In ColE1-type plasmids, RNA I (the ctRNA) base-pairs with RNA II (the replication primer precursor), aborting replication initiation (Fig. 1b)^22^. RNA I is produced at 100-fold higher levels than RNA II and has fast turnover with a half-time of 1,3 to 2 min^23^. The plasmid-encoded Rom (Rop) protein, an auxiliary factor adjacent to the minimal replicon, stabilizes the RNA I-RNA II duplex, increasing repression efficiency^4^. RNA polyadenylation by PcnB further accelerates RNA I decay; deletion of *pcnB* extends RNA I half-time ( > 15 min) and lowers PCN^24^. In the IncFIIA plasmid R1 from *Salmonella*, the antisense RNA molecule CopA binds to the CopT leader sequence of *repA* mRNA, blocking translation of RepA, the rate-limiting initiator protein (Fig. 1b upper). Approximately 20 RepA molecules are required to initiate replication at the *oriR1* site^25,26^. In addition to the CopA/CopT system, R1 encodes CopB, which is produced from the constitutive *pcopB* promoter on the long *repA* transcripts and forms tetramers that bind and repress the *prepA* promoter, preventing excess *repA* expression at normal copy number. When PCN drops, CopB reductions relives *prepA* repression, triggering increased *repA* transcription and replication in plasmid-poor cells. This auxiliary feedback complements CopA’s antisense block, ensuring rapid recovery from low-copy states and minimizing plasmid loss during cell division^27,28^ (Fig. 1b lower).
- **Combined antisense RNA and protein control**: Some replicons integrate antisense RNA with protein repressors for fine tuning of PCN control^22^. In *Streptococcus*, plasmid pMV158 uses RNAII to block RepB translation and CopG repressor protein to repress *repB* transcription. This dual mechanism corrects both sudden and gradual PCN fluctuations, ensuring robust stability (Fig. 1c)^4,20^.
- **Rep-only mechanism**: Some small plasmids lack both iterons and antisense RNAs. Instead, they rely on Rep protein autoregulation, excess Rep binds nonproductively to origin elements, preventing further replication initiations^29^.

### Other mechanisms of plasmid maintenance

While replication control sets PCN, two additional systems (active partitioning and post-segregational killing) ensure that plasmid copies are reliably inherited during cell division^7,30^. Additionally, mechanisms that resolve plasmid multimerization also play a crucial role in maintaining plasmid stability.

- **Active partitioning**: Bacterial plasmids often encode one or more partition systems. They typically comprise a cis-acting centromere-like DNA sequence and a trans-acting par operon, which encodes two proteins: a centromere-binding protein (CBP) that forms the partition complex at the centromere site, and a NTPase that interacts with this complex to drive plasmid segregation. The assembly of a nucleoprotein complex at a centromere-like site on the plasmid facilitates proper segregation and therefore guarantees that each daughter cell receives at least one plasmid copy after division, which is especially critical for low-copy-number plasmids. Three main classes of plasmid partition systems have been described.
  i. Type I (ParABS), commonly found in low-copy-number plasmids such as P1 and F, as well as in bacterial chromosomes, includes a Walker-type ATPase (ParA), a CBP (ParB), and a centromere-like site (ParS).
  ii. Type II (ParMRC), identified in *Escherichia coli* R1 plasmids, encodes an actin-like ATPase (ParM) and a CBP (ParR), which also plays a role in transcriptional autoregulation of the operon.
  iii. Type III (TubZRC), found in plasmid pXO1 of *Bacillus anthracis*, encodes a tubulin-like GTPase (TubZ) and a helix-turn-helix (HTH) domain-containing CBP (TubR).
    By actively partitioning, these systems reduce reliance on high PCN for stable inheritance^7,30^.
- **Post-segregation killing (PSK**): Toxin-antitoxin (TA) systems are also common widespread PSK systems that secure plasmid maintenance by eliminating or inhibiting rare plasmid-free daughter cells^30^. TA loci are classified into seven types (I–VII) based on the nature and action of the antitoxin. The type II system is the most common TA system, where each operon encodes a stable protein toxin and a more labile protein antitoxin. The antitoxin neutralizes the toxin by tight complex formation and autoregulates the TA operon via its N-terminal DNA-binding domain. When a cell fails to inherit the plasmid, its residual antitoxin is rapidly degraded by host proteases (e.g. Lon, Clp), freeing the stable toxin to arrest growth or kill the cells. There are eight superfamilies of Type II TA systems: RelBE, MazEF, VapBC, CcdAB, ParDE, HigAB, HipBA, and Phd-Doc. Briefly, VapBC (toxin = VapC endonuclease; antitoxin = VapB) is especially prevalent on virulence plasmids of *Salmonella* and *Shigella*, where it both enforces PSK and enhances intracellular fitness. CcdAB (toxin = CcdB gyrase inhibitor; antitoxin = CcdA) is conserved on F‑type and several enterobacterial virulence plasmids, although in some lineages (e.g. *Salmonella enterica* serotype *Typhimurium* pSLT) CcdB is inactivated, and the locus may serve regulatory rather than killing functions. GNAT‑type TA modules (GmvAT) acetylate tRNAs to arrest translation and likewise contribute to plasmid stability in *Shigella* and enterohemorrhagic *E. coli*. ParE-type highly abundant in both plasmid and chromosome and known to maintain the broad range plasmid IncP RK2.
  Recent studies reveal that the ParDE toxin–antitoxin system reduces PCN under ParE stress and directly tunes replication^31^. The ProT/PrpA pair also limits copy number when ProT overproduction threatens cell viability, as PrpA binds and neutralizes ProT, autoregulates its own operon and blocks RepB binding at iteron repeats. By interfering with RepB–ori interactions, PrpA prevents over-replication^32^. These examples show that TA systems not only maintain plasmids but also actively adjust copy number during stress.
- **Plasmid multimerization and resolution:** Plasmids face inherent challenges related to their complex structural dynamics, as the formation of plasmid multimers can compromise their stable segregation. General host-mediated homologous recombination, often relying on host *rec* genes, can lead to the formation of plasmid multimers (e.g., dimers), particularly in multicopy plasmids, which can impede proper segregation^33^. Conversely, some plasmids, notably low-copy ones like P1, encode dedicated site-specific recombination systems that actively resolve these multimers back into monomeric units, thereby ensuring stable inheritance^34^. Collectively, the balance between multimer formation and their resolution critically affects the effective PCN available for stable inheritance, particularly for low-copy plasmids.

### Genetic features shaping steady-state PCN

With the growing availability of complete plasmid sequences from diverse bacterial species and ecological niches, researchers can now tackle broad questions about the genetic determinants of PCN, such as the influence of plasmid length, replicon multiplicity, host range, and accessory gene load. Ramiro-Martínez et al.^35^ analyzed over 6000 plasmids and found that intrinsic replication-control circuits maintain PCN robustly across hosts and genetic backgrounds, with only a handful of replicon types and PTUs exhibiting host-dependent variation. Notably, plasmid size emerged as the strongest negative predictor of PCN. Similarly, Maddamsetti et al.^36^ applied the PIRA pipeline to more than 11,000 plasmids, and identified a universal inverse power-law relationship between PCN and plasmid length, where small plasmids clustered at ~28 copies per cell, whereas large plasmids overlap chromosomal replication patterns at ~1–2 copies. This pattern highlights fundamental biophysical constraints on plasmid dosage. Together, both studies show that while replication controls keep PCN stable across different hosts and gene contents, but basic physical limits, especially plasmid size, dictate intrinsic upper and lower PCN limits. Importantly, these bioinformatic estimates reflect steady-state averages; capturing PCN dynamics under fluctuating environmental conditions will require dedicated experimental approaches.

## PCN and bacterial fitness

Even though bacteria have evolved regulatory mechanisms to minimize plasmid burden, carrying extra plasmid copies still imposes a fitness cost primarily due to the expression of plasmid-associated traits. This was illustrated in a study where in the absence of antibiotic selection, antibiotic resistance was lost from bacterial populations despite high PCN being maintained. This was attributed to the rapid evolution of “stealth plasmids”, which are variants that had deleted their costly AMR genes. Those plasmids retained high PCN with lower fitness cost, outcompeted and transmitted more effectively, ultimately displacing the full AMR plasmids in the bacterial population^37^. This highlights how PCN lies at the heart of this cost-benefit trade-off: higher PCN boosts plasmid-encoded functions like antibiotic resistance thus often increases metabolic demand, whereas lower PCN reduces the burden from gene expression but risks plasmid loss. Indeed, each additional plasmid copy consumes nucleotides, replication factors, and energy, slowing bacterial growth^21^. In tunable ColE1 system, bacterial growth rate declined across a 1–800 copies range, with a 0.063% reduction per copy and a rise in non-growing cells at high PCN. Yet, plasmids persist owning to their selective advantages and stable maintenance systems^38^.

Raising PCN to around 60 in *E. coli* altered metabolism: accelerating RNA and protein turnover, reducing mRNA stability, and lowering translation efficiency, especially at intermediate PCN^39,40^. High PCN also drives overproduction of replication initiators, delaying chromosomal replication, triggering the SOS response, and inhibiting cell division in both *E. coli*^41^ and *Pseudomonas aeruginosa*^42^. In *Klebsiella pneumoniae* and *E. coli*, each additional kilobase of plasmid DNA imposes an in vitro fitness cost of approximately 2.3 × 10^-5^. Though this cost is lower in vivo, this cost is outweighed under antibiotic pressure and during infections, where high PCN enhances resistance^43^. Paradoxically, multicopy plasmids can also enhance evolvability by maintaining both ancestral and mutated alleles within the same cell, which delay the loss of less-fit variants and preserve genetic diversity under fluctuating selection^44^. These examples illustrate how PCN balances metabolic cost with adaptative potential, and why bacteria invest heavily in precise mechanisms to regulate and maintain plasmid dosage. These mechanisms represent critical factors in the emergence and persistence of antibiotic resistance plasmids in bacterial populations.

## PCN variation as a bacterial virulence strategy

Virulence functions in many pathogenic bacteria are encoded on large ( > 50 kb), low-copy-number plasmids. However, recent studies reveal that the copy number of virulence plasmids is not static; it can vary during infection, and in some cases, increase pathogenicity. In *Yersinia pseudotuberculosis*, PCN has been identified itself as a novel virulence mechanism. Wang et al.^45^ showed that the IncFII-class virulence plasmid, which encodes the type III secretion system (T3SS)–essential for host cell contact and toxin translocation–increased its copy number during infection. Under T3SS-inducing conditions (37 °C, low Ca^2+^), PCN increased up to threefold compared to the T3SS-repressing conditions (26 °C with Ca^2+^), amplifying the dosage of all plasmid-encoded genes.

Genetic dissection revealed that this temperature-dependent PCN increase occurred in a Δ*yopD* mutant but not in a Δ*lcfF* mutant, indicating YopD’s role as a negative regulator of plasmid amplification. Overexpression of CopA (antisense RNA modulating RepA translation) reduced PCN under T3SS-inducing conditions and alleviated associated growth and expression burdens. Importantly, engineered strains with fixed low PCN showed impaired T3SS expression, reduced host cell translocation, and attenuated virulence in a mouse model, while restoring plasmid dosage reversed these phenotypes^46^. These findings highlight PCN as an adaptative mechanism by which *Yersinia* modulates virulence gene expression in response to host signals, balancing pathogenic potential with metabolic cost.

Further ddPCR-based analysis of PCN dynamics in vivo showed PCN of *Y. pseudotuberculosis* populations varies spatiotemporally across different tissues during murine infection^47,48^. PCN peaked at approximately 6 copies per cell one day post-infection, then gradually declined to around 2.5 copies as infection progressed. Organs such as the Peyer’s patches and cecum, early colonization sites, showed higher PCN, whereas deeper tissues such as mesenteric lymph nodes, spleen, and liver displayed lower PCN^48^. These findings highlight the importance of elevated PCN during the initial clonal expansion and suggest a dynamic regulatory mechanism that is finely tuned to tissue-specific environmental cues. Recent studies implicate YmoA, YopD and PcnB/PAPI in sensing environmental signals to modulate PCN, virulence gene expression, stress responses and metabolic output during Yersinia pathogenesis^49^.

The idea of PCN variation as a virulence strategy is gaining interest, supported by similar findings in other pathogens such as *Agrobacterium tumefaciens* and *Sinorhizobium fredii*^50,51^. Evidence suggests that this regulatory mechanism enabling rapid adjustments of plasmid-encoded functions in response to environmental cues, is likely a widespread strategy among plasmid-bearing bacteria.

In *Salmonella enterica* serovar Typhimurium, the low-copy-number conjugative IncF virulence plasmid pSLT also exhibits substantial cell-to-cell PCN variability. Using a fluorescent repressor–operator tagging system to visualize pSLT in live cells grown in fresh LB or mouse serum (to mimic host conditions), researchers observed PCN ranging from one to eight per cell in mid-exponential phase. This heterogeneity translated into different expression of plasmid-encoded virulence determinants, suggesting that subpopulations with elevated PCN might enhance conjugation rates and infection potential^52^. Despite the presence of a plasmid partitioning system, the basis of this variability remains unresolved.

In contrast, stress conditions can drive PCN down. Ruan and Bourne^31^ demonstrated this using an arabinose-inducible ParE1 toxin encoded on a ColE1-like plasmid. When PCN was high, cells succumbed to lethal doses of ParE toxin; reducing PCN allowed survival under sublethal toxin exposure. However, lowering PCN below a critical threshold compromised viability, as essential plasmid-borne genes became under-represented. Remarkably, bacteria tolerated ParE toxicity primarily by actively reducing plasmid burden, revealing a novel adaptive mechanism. Evolved clones also acquired mutations in DNA polymerase I, suggesting a conserved evolutionary route to stabilize low PCN under toxic stress.

## PCN as a modulator of antibiotic resistance

PCN directly modulates the dosage of antibiotic-resistance genes. Resistance can be elevated either by genetic changes that raise PCN or by antibiotic exposure that transiently amplifies PCN without mutation. This section first examines mutation-driven increases in PCN, then explores antibiotic-induced PCN modulation, and finally discusses PCN’s role in antibiotic heteroresistance.

### Mutation-driven increase in PCN

Under constant antibiotic selection, mutations in plasmid replication control genes can elevate PCN, thereby increasing resistance. For instance, *E. coli* 600 carrying a *bla*_*NDM-5*_-positive IncX3 plasmid, evolved under meropenem selection, acquired a GAT → TAT point mutation in *repA*. This inactivated RepA’s autorepression and raising both PCN and conjugation frequency^53^. Similarly, in a patient’s gut during antibiotic treatment, selection for high-copy-number pOXA-48 plasmid variants was observed, often linked to mutations upstream of the *repA* gene that significantly elevated PCN, AMR but reduced fitness^54^. San Millan et al. reported that mutations in the RNAI, responsible for the control of plasmid replication, led to the evolution of ceftazidime resistance and concomitant increased PCN of a small multicopy pBGT plasmid^55^. Another key example comes from R1 plasmid-bearing populations evolved under antibiotic stress repeatedly generated parallel mutations in *copA*, disrupting the CopA antisense RNA structure and derepressing replication, ultimately increasing PCN, MICs, and transfer rate^56^. In broad-host-range IncP-1 plasmids, adaptation in diverse bacterial hosts resulted in *trfA* duplications or N-terminal truncations that elevated PCN, enhancing plasmid stability and host fitness, whereas frameshift variants did not^57^. In *E. coli* evolved with the pBGT multicopy plasmid (19 copies per cell) carrying a TEM-1 gene, resistance to ceftazidime rose roughly 10-fold, driven by mutations in the *oriV* that disrupted the loop region of the RNAI-RNAII interaction site, releasing replication repression^55^.

Not all plasmids respond to antibiotics by increasing PCN. For instance, an unstable ColE1-derived vector (pGFPuv) under carbenicillin selection showed decreased PCN due to segregation instability^58^. Conversely, a recent study reported that PCN rose after antibiotic exposure in wildtype strains, while strains lacking the *pcnB* showed reduced PCN and increased antibiotic susceptibility^59^. There is another very recent study showed *pcnB* in *Yersinia* and *Shigella* promotes virulence plasmid (IncFII type) stability, suggesting pcnB/PAP I as a potential drug target^60^.

### Antibiotic-induced PCN modulation

Antibiotic exposure can transiently boost PCN without detectable mutations in replication genes. In *Staphylococcus aureus*, insertion of small *lnu(A)-*carrying plasmids increased PCN and lincosamide resistance upon drug exposure, despite no mutations in *rep* sequences^61^. Similarly, *Haemophilus influenza* transformed with a *Pasteurella multocida* plasmid exhibited up to 44-fold PCN increases under ampicillin pressure; whole-genome sequencing ruled out replication gene mutations^62^. Single-cell tracking and simulations using a high PCN (10-30 copies per cell) *bla*_TEM-1_ plasmid in *E. coli* showed that antibiotic selection amplifies pre-existing PCN heterogeneity. Bacterial cells with higher PCN preferentially survived, shifting the population distribution and enabling rapid, transient resistance without requiring mutations^63^. Similar PCN heterogeneity was also observed in low PCN plasmids (1-8 copies per cell), leading to differential expression of plasmid-encoded genes among subpopulations^52^.

### PCN and antibiotic heteroresistance

Heteroresistance (HR), the coexistence of bacterial subpopulations with different antibiotic susceptibility, is an escalating clinical problem observed in various bacterial species and can reduce treatment efficacy across diverse antibiotic classes^64^. Among clinical Enterobacterales, 27.4% exhibit heteroresistance^65^. Three concurrent mechanisms underline this phenomenon: tandem gene amplification, increased PCN and transposition of resistance genes onto cryptic plasmids^43^. A detailed case in *K. pneumoniae* clinical isolate demonstrated HR phenotype towards 5 antibiotics (amikacin, AMK; tobramycin, TOB, ertapenem, ETP; tigecycline, TGC, and ceftazidime: avibactam, CZA). A 3- to 89-fold PCN increases of a low-copy number IncFIB plasmid (p96) was observed after antibiotic exposure. Meanwhile, a Tn3 transposon carrying ARGs transposed from p96 into a cryptic ARG-free plasmid, followed by an increase of PCN in the cryptic plasmid up to 172 copies (TPCN). It is suggested that antibiotic-induced transposition and plasmid multimerization creates plasmid repeats that are subsequently expanded in a RecA-mediated recombination. These changes amplified gene dosage and resistance, while also demonstrating the evolutionary flexibility of small cryptic plasmids. Both the elevated PCN and TPCN were highly prevalent among *E. coli* bloodstream infection (BSI) isolates and were detected in vivo using a mouse gut colonization model, indicating their highly clinical relevance in HR and AMR emergence^43^. Additionally, Pal & Anderson^66^ observed that plasmid-borne resistance genes underwent significantly greater copy-number amplification under antibiotic stress than their chromosomal counterparts, further underscoring the impact of genetic location on heteroresistance and resistance evolution, indicating their clinical relevance in the emergence of HR and AMR.

## Evolutionary dynamics of PCN

Beyond immediate costs and benefits, PCN plays a fundamental role in shaping long‑term evolutionary trajectories by modulating mutation supply, genetic drift, and host–plasmid co‑adaptation. First, adaptive mutations typically arise in the host chromosome rather than the plasmid itself, as the bacterial genome ( ~ 4.6 Mb) offers a vastly larger mutational target^67^. These chromosomal compensatory mutations are frequently more effective at mitigating the fitness costs of plasmid carriage than plasmid-borne mutations. This process establishes compensated bacterial lineages as stable “hubs” for plasmid accumulation and dissemination^68^. Such adaptations stabilize plasmid maintenance by fine-tuning replication or partitioning systems without directly altering *rep* loci^69–71^.

Second, high PCN simultaneously boosts mutational supply and amplifies segregational drift, a process where plasmid copies are randomly partitioned during cell division. This process slows the fixation of new alleles and promotes within-cell allele diversity, a state known as plasmid heteroplasmy^67^. Ilhan et al.^67^ first demonstrated that high PCN increases mutational input, but segregational drift delays fixation, maintaining heteroplasmy. Garoña et al.^72^ confirmed that multicopy plasmids experience stronger drift constraints than haploid chromosomes, limiting their effective evolutionary rate under weak selection. Bedhomme et al.^73^ demonstrated that heteroplasmy sustains clonal interference, where competing plasmid variants coexist, thereby slowing down selective sweeps. Dewan & Uecker^74^ further quantified this, showing that segregational drift establishes a threshold at which only beneficial alleles with sufficient selective advantage can persist on high-PCN plasmids. However, not all plasmid mutations contribute equally to adaptation. Rodríguez-Beltrán et al.^75^ revealed that genetic dominance interacts with high PCN to shape evolutionary outcomes: recessive mutations are masked by wild-type copies, while dominant mutations are expressed immediately and are more likely to become fixed. Crucially, San Millán et al.^55^ further showed that under strong positive selection, this dynamic shifts to high PCN not only increases mutational supply but also amplifies the phenotypic impact of dominant gain-of-function mutations via gene dosage, significantly accelerating the evolution of clinically relevant resistance. Finally, chromosomal compensatory evolution stabilizes the host–plasmid relationship by offsetting plasmid fitness costs without disrupting plasmid replication or transfer functions, as shown by Wright et al.^68^. This host-level buffering preserves the plasmid’s ecological potential and, when elevated gene dosage is beneficial, can help maintain high-PCN states under selection^70^.

Collectively, these studies illustrate how high PCN can both constrain and accelerate adaptation through the intricate interplay of mutation supply, genetic drift, dominance, and dosage. Taken together, processes such as host adaptation, heteroplasmy, within-cell clonal interference, and selection for gene dosage reveal that PCN is an evolvable trait, finely tuned by the balance between mutation, segregational drift, and positive selection. However, the relative contributions of these factors are likely context-dependent, varying across species, plasmid types, and ecological conditions.

## Concluding remarks

PCN remains largely overlooked yet serves as a master regulator of bacterial adaptation, controlling the expression of resistance genes and virulence factors through dynamic feedback circuits—iteron repeats, antisense RNAs and auxiliary protein repressors. By tightly controlling PCN, bacteria balance the metabolic burden of extra DNA with the need for elevated gene dosage under stress.

Crucially, increased PCN also enhances plasmid conjugation rates, accelerating the spread of antibiotic resistance genes and virulence determinants. This process fuels the emergence of multidrug-resistant pathogens and hypervirulent strains in both clinical and environmental settings. Modern integrated approaches, including experimental evolution, single‑cell resolution measurements, and comparative genomics will begin to disentangle PCN heterogeneity during infection, with potential to uncover biomarkers for detecting heteroresistance and guiding personalized therapy. Despite its importance, PCN has received limited attention. Further research into its regulatory mechanisms (iteron architecture, antisense RNA dynamics and auxiliary feedback proteins) will be essential. Ultimately, targeting PCN control could disable resistance- and virulence-bearing plasmids, offering a promising anti-replication strategy against future pandemic threats.

## Data Availability

No datasets were generated or analysed during the current study.
